# Health-related quality of life in rural cancer survivors compared with their urban counterparts: a systematic review

**DOI:** 10.1007/s00520-024-08618-9

**Published:** 2024-06-12

**Authors:** S. Latham, M. J. Leach, V. M. White, K. Webber, M. Jefford, K. Lisy, N. Davis, J. L. Millar, S. Evans, J. D. Emery, M. IJzerman, E. Ristevski

**Affiliations:** 1 Department of Oncology, Monash Health, Clayton, Victoria Australia; 2 Department of Oncology, Eastern Health, Box Hill, Victoria Australia; 3 Medical Oncology, Alfred Health, Melbourne, Victoria Australia; 4 School of Rural Health, Monash University, Bendigo, VIC Australia; 5 School of Psychology, Deakin University, Melbourne, Victoria Australia; 6 School of Clinical Sciences, Monash University, Clayton, Victoria Australia; 7 Department of Health Services Research, Peter MacCallum Cancer Centre, Melbourne, Victoria Australia; 8 Australian Cancer Survivorship Centre, Peter MacCallum Cancer Centre, , Melbourne, Victoria Australia; 9 Sir Peter MacCallum Department of Oncology, University of Melbourne, Melbourne, Victoria Australia; 10 Cancer Survivor, Melbourne, Australia; 11 Central Clinical School, Monash University, Melbourne, Victoria Australia; 12 Radiation Oncology, Alfred Health, Melbourne, Victoria Australia; 13 Victorian Cancer Registry, Cancer Council Victoria, Melbourne, Victoria Australia; 14 School of Public Health and Preventive Medicine, Monash University, Melbourne, Victoria Australia; 15 Department of General Practice and Primary Care, Melbourne Medical School, University of Melbourne, Melbourne, Victoria Australia; 16 Centre for Cancer Research, Melbourne Medical School, University of Melbourne, Melbourne, Victoria Australia; 17 Centre for Health Policy, Cancer Health Services Research, Melbourne School of Population and Global/Total Health, The University of Melbourne, Carlton, Victoria Australia; 18 School of Rural Health, Monash University, 15 Sargeant Street, Warragul, VIC 3820 Australia

**Keywords:** Cancer survivors, Health-related quality of life, Rural, Urban

## Abstract

**Purpose:**

We conducted a systematic review to describe health-related quality of life (HRQOL) in rural cancer survivors (RCS), and compare HRQOL between RCS and urban cancer survivors (UCS).

**Method:**

We searched Medline, Embase, CINAHL Plus, and PsycINFO for studies with HRQOL in adult cancer survivors living in rural, regional, remote, and urban areas, who had completed definitive primary cancer treatment, without evidence of residual disease. Where available, we used normative and clinically important values to ascribe meaning to HRQOL data.

**Findings:**

Fifteen studies (16 papers) were included. Most were from the US (*n* = 8) and reported on breast cancer survivors (*n* = 9). Six HRQOL instruments, collecting data across 16 domains, were used. Three instruments were specific to the survivorship phase. Normative and clinical data were available for 12 studies. Compared with normative populations, RCS had clinically worse physical HRQOL (6/12 studies), better social/family (5/7), and functional (3/6) HRQOL, and there were no differences in emotional or/mental HRQOL (9/12). In six studies with rural–urban comparator groups and normative and clinically important data, RCS and UCS had clinically worse physical (3/6 and 2/6, respectively) and better social/family (3/4 and 2/4 studies, respectively) HRQOL than normative populations. Functional HRQOL was better in RCS (2/4 studies) than UCS and normative populations. In 3/6 studies, there were no clinical differences in emotional or/mental HRQOL between RCS, UCS, and normative populations.

**Conclusion:**

Overall, HRQOL is not clearly better or worse in RCS than UCS. Future research should include different tumor types, rural residents, and survivorship-specific HRQOL instruments.

**Supplementary Information:**

The online version contains supplementary material available at 10.1007/s00520-024-08618-9.

## Introduction

With more people living beyond a cancer diagnosis [1], a focus on quality of life (QOL) in the post-treatment survivorship phase is imperative [2]. The long-term impacts and late effects of cancer and its treatment persist post-treatment, as does the need to address supportive care needs across all facets of QOL [3–5]. The most comprehensive and cross-cultural definition of QOL is the World Health Organization’s (WHO’s) definition: “an individual’s perception of their position in life in the context of the culture and value systems in which they live and in relation to their goals, expectations, standards and concerns” [6]. Therefore, QOL consists of multiple domains, including physical, psychological, level of independence, social, relationships with the environment, and spiritual/religious/personal beliefs [7]. Alongside QOL, health-related QOL (HRQOL) has also been increasingly used in healthcare research and clinical practice [8, 9]. Defining HRQOL is complex; there are multifarious definitions and measures of HRQOL. Often, QOL and HRQOL have been used interchangeably and can be difficult to disentangle from each other [10]. In this article, HRQOL refers to “the health aspects of quality of life, generally considered to reflect the impact of disease and treatment on disability and daily functioning; it has been considered to reflect the impact of perceived health on an individual’s ability to live a fulfilling life” [11].

While HRQOL has been examined broadly in cancer survivors [12–15], or in specific tumor types [16–20], there is no consolidated evidence on HRQOL in rural, regional, or remote (hereafter referred to as rural) cancer survivors (RCS), or HRQOL comparisons between RCS and urban cancer survivors (UCS). Systematic review evidence suggests that rurality negatively impacts cancer survival [21, 22]. Whether this is the case for HRQOL is unclear. We conducted a systematic review to describe RCS’ post-treatment HRQOL, as measured using validated scales, and investigate if there were any similarities or differences in global or domain-specific HRQOL between RCS and UCS and normative populations. Our review is important as it establishes the known evidence base and provides direction for future research, hence avoiding duplication of unnecessary primary research. People in rural areas want to see research which informs policy and provides a clear evidence-base for health care change. Consolidating knowledge on HRQOL will help clinicians, service providers, and cancer control authorities identify what supports are needed to improve HRQOL among cancer survivors, and how these supports may differ by geographical location.

## Methods

Our systematic review protocol was registered with PROSPERO (registration number: CRD42021219769). The methods have been reported in accordance with the Preferred Reporting Items for Systematic Reviews and Meta-Analyses guidelines [23].

### Search strategy

We searched Ovid Medline, EMBASE, CINAHL Plus, and PsycINFO databases for studies published in English between 1 January 2000 and 13 July 2023. We used the same keywords across databases to maintain consistency. Keywords focused on three topic areas: population (cancer, neoplasms, survivor*, post-treatment), location (rural*, regional*, remote*, non-urban, non-metropolitan, urban, metro*, non-rural), and outcome (Quality of life, well-being, life satisfaction). See Supplementary Material 1 for the search strategy.

### Eligibility criteria

Studies with cancer survivors aged at least 18 years at diagnosis and residing in rural, regional, or remote areas, with or without urban or metropolitan counterparts, were included. While a cancer survivor has been broadly described from the time of diagnosis to end of life [24], for the purpose of this review, we focused on individuals who had completed definitive treatment and demonstrated no evidence of residual or recurrent disease. Individuals receiving adjuvant endocrine therapy for breast cancer were included. Each study’s area(s) of residence were checked against the relevant country’s national classification systems or census definitions. In instances where no classification or definition was provided, the authors’ definition was used. Studies were excluded if the study did not specifically report RCS’ HRQOL. Only studies that used a validated HRQOL instrument or survey were included. Studies were excluded if they reported on only one HRQOL domain. Papers describing study protocols and literature/systematic/scoping reviews, conference abstracts, editorials, and opinion pieces were excluded.

### Screening and data extraction

One author (ER) performed database searches, imported papers into EndNoteX9 (Clarivate, Philadelphia, PA, USA), and removed duplicates. Remaining papers were imported into Covidence (Veritas Health Innovation Ltd, Melbourne, AUS) for screening and management. Three reviewers (ER, SL, VW) independently screened article titles and abstracts. Full-text papers were screened independently; ER screened all papers and SL, VW, and KW screened one-third each. Any discrepancies were discussed by reviewers until consensus was reached. Two reviewers (ER, SL) extracted data from included studies. Data extracted included authors, publication year, country, study design, recruitment method, area of residence, sample size, participant characteristics (age, sex, cancer type, and time since diagnosis), HRQOL instrument, and HRQOL summary statistics. For randomized or non-randomized controlled trials, and for pre- and post-intervention studies, only participants’ baseline characteristics and HRQOL summary statistics were extracted, as the aim of the review was not to assess intervention outcomes. Data extraction and management were undertaken in Excel (Microsoft, Seattle, WA, USA).

### Quality assessment

Three Johanna Briggs Institute (JBI) critical appraisal tools were used to assess the methodological quality of studies: randomized controlled trials [25], quasi-experimental studies [26], and analytical cross-sectional studies [26]. Each tool contains a checklist of questions which are assessed as “yes,” “no,” “unclear,” or “not applicable.” The quality of included studies was reported as “moderate” or “high” if they met ≥ 50% or ≥ 75% of eligible quality criteria, respectively. All studies meeting inclusion criteria were included in the review due to the emergent nature of this research topic. Quality appraisal was conducted independently by two reviewers (ER, SL) and any discrepancies were discussed until consensus was reached.

### Data synthesis and analysis

We examined whether there were statistically and clinically significant differences in HRQOL between study participants and normative populations. Comparing study and normative data is useful for benchmarking individuals/groups against what is considered typical or average [27]. However, as statistically significant differences may not imply real-world importance, we also examined clinically significant differences in mean HRQOL scores between study and normative populations [27–29].

### Statistical significance

To examine statistically significant differences in HRQOL between study participants and normative populations, we calculated or sourced 95% confidence intervals (CIs) for each domain in each study and assessed any cross-over with the corresponding study and normative 95% CIs [27, 28]. If there was no cross-over in 95% CIs, then we considered it a statistically significant difference. We used normative data relevant to the HRQOL instrument and where possible country-specific values. For the Functional Assessment of Cancer Therapy-General (FACT-G) instrument, we used US- [30] and Australian- [31] based normative values. For the Medical Outcomes Study Short-Form 12 or 36 (SF-12 or SF-36) and European Organisation for Research and Treatment of Cancer Quality of Life Core-30 questionnaire (EORTC QLQ-C30) instruments, we used US-based values [32, 33].

### Clinical significance

To examine clinically significant differences in HRQOL between study participants and normative populations, we used anchor-based approaches for minimum clinically important difference (MCID) [30, 34] or thresholds for clinical importance (TCI) [35] relevant to each HRQOL instrument.

#### MCID anchors

For studies with MCID anchors, we subtracted the mean normative score from the mean study score and compared the difference with recommended MCID values. On the FACT-G, differences of five or more points in mean global scores, and two or more points in mean domains scores, indicated clinical significance [30]. On the SF-12 or SF-36, a difference of five or more points on the mean physical and mental component summary scores indicated clinical significance [34]. To examine the direction of HRQOL, negative scores indicated HRQOL was worse in the study population than the normative population and positive scores indicated HRQOL was better in the study population compared to the normative population.

#### TCI anchors

For studies with TCI anchors, a mean study score less than the TCI anchor on the functioning scales on the EORTC QLQ-C30 (physical, role, social, emotional, cognitive) indicated a clinically important problem (i.e., worse HRQOL), while a mean study score greater than or equal to the TCI anchor was clinically unimportant (i.e., better HRQOL) [35].

### Studies without normative data and clinical anchors

For studies without normative data and clinical anchors, we extracted the mean HRQOL scores for each domain and examined whether the scores indicated high or low HRQOL on each domain. We described these results narratively.

### Rural–urban comparisons

For studies with rural and urban participants, we firstly extracted the reported results of multivariable analyses between RCS and UCS to examine if there were any differences between rural and urban participants in each study. We then used the analysis methods described above to determine if the HRQOL of study participants (RCS and UCS) was clinically significantly different from normative populations.

## Results

### Study selection

**Fig. 1:**
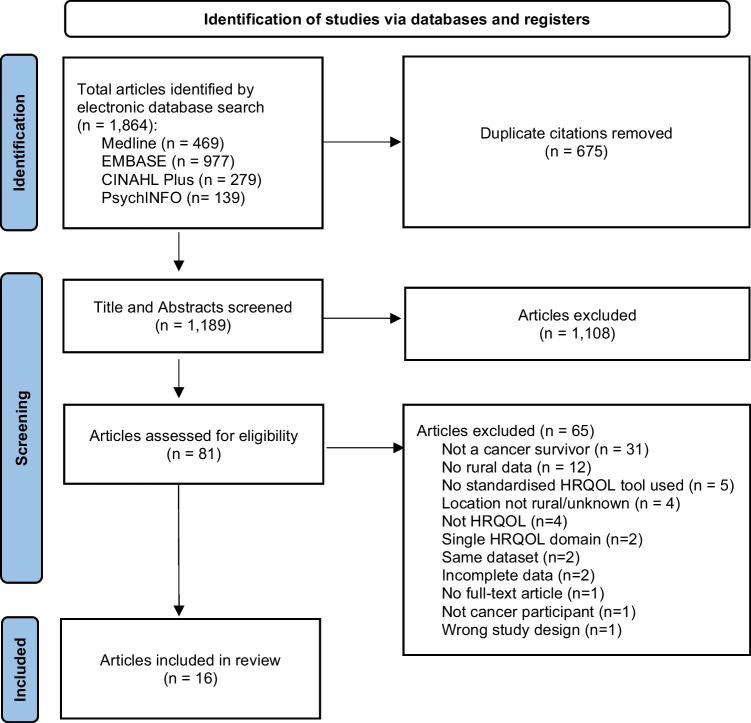
PRISMA flow diagram describing selection of studies for inclusion in the review

We identified 1864 unique papers through the database searches. After removing 675 duplicates, 1189 titles and abstracts were screened. Of these, 16 papers (reporting data from 15 unique studies) were included in our review (Fig. 1). Two papers [31, 36] used the same dataset but reported on different participant subgroups: they are substudies of the one study.

### Participant and study characteristics

Participant and study characteristics are reported in Table 1. Of the 16 papers, eight were from the United States (US) [37–44], with the remaining from Australia [31, 36, 45], Ireland [46, 47], Canada [48], Pakistan [49] and Poland [50]. Participants were mainly recruited through cancer registries (*n* = 7) or regional or community cancer centers and hospitals/local clinics (*n* = 7). Government-based classification systems were used to define rurality (*n* = 9).

**Table 1:** Study, participant, and health-related quality of life characteristics (*n*** = **16 papers)

(Reference number) Author, publication year	Country of study	Study design	Study aim	Study recruitment method/s and period	Rural/urban classification system	Cancer type/s	Total participants *n* Rural *n* (%) Urban *n* (%)	Female participants *n* (%)	Years since diagnosis (% or m ean/SD)	HRQOL instrument used: domains reported
[49] Azam et al. 2021	Pakistan	Cross-sectional	Assessed HRQOL in relation to age, marital status, marital age, and family history of breast cancer, breast feeding, abortion and still birth	Recruitment through one hospital between January and December 2020	No specific classification reported	Breast	250 94 (37.6%) 156 (62.4%)	250 (100%)	Not stated	QOL-Breast Cancer: physical, psychological, spiritual, total score
[47] Cahir et al. 2017	Ireland	Cross-sectional	Examined the association between HRQOL and treatment-related symptoms in breast cancer survivors prescribed endocrine therapy	Recruitment through the National Cancer Registry Ireland between 1 July 2009 and 30 June 2014	Composite measure: settlement size, population density, and proximity to the patient’s treatment center	Breast	1568 698 (44.5%) 870 (55.5%)	1568 (100%)	1–5 (100%)	FACT-G (with FACT-ES): p hysical, emotional, social/family, functional, total score, endocrine symptoms
[36] DiSipio et al. 2009 †	Australia	Cross-sectional	Examined demographic and clinical correlates of HRQOL in rural and regional breast cancer survivors	Recruitment through Queensland Cancer Registry between 2002 and 2007	Accessibility/Remoteness Index of Australia (ARIA)	Breast	323 323 (100%) 0 (0%)	323 (100%)	1 (100%)	FACT-G (with FACT-B + 4): physical, emotional, social/family, functional, total score, breast cancer symptoms, arm symptoms
[31] DiSipio et al. 2010 †	Australia	Cross-sectional	Examined demographic and clinical correlates of HRQOL in urban and non-urban cancer survivors	Recruitment through the Queensland Cancer Registry between 2002 and 2007	Accessibility/Remoteness Index of Australia (ARIA)	Breast	600 323 (53.8%) 277 (46.2%)	600 (100%)	1 (100%)	FACT-G (with FACT-B + 4): physical, emotional, social/family, functional, total score, breast cancer symptoms, arm symptoms
[37] Fazzino et al. 2018	United States	Retrospective, pre-post	Assessed the impact of a weight management intervention on physical activity, late effect symptoms of breast cancer, and weight management	Recruitment through 11 community cancer centers and clinics in three US states between October 2011 and September 2013	Rural/urban community area (RUCA)	Breast	176 176 (100%) 0 (0%)	176 (100%)	Not stated	SF-12: p hysical, mental
[38] Gray et al. 2019	United States	Randomized controlled trial	Examined a home-based diet and exercise intervention (Reach-out to ENhancE Wellness [RENEW]) in cancer survivors aged ≥ 65 years	Recruited through North Carolina Central and Duke cancer registries and physician referrals between 1 July 2005 and 17 May 2007, July 2006 and May 2008, and July 2007 and May 2009	Rural/urban community area (RUCA)	Breast, colorectal, prostate	487 160 (32.9%) 327 (67.1%)	269 (55.2%)	≥ 5 (100%)	SF-36: p hysical, mental
[45] Mandaliya et al. 2016	Australia	Cross-sectional	Examined the association between financial stress and HRQOL in rural cancer survivors	Recruitment through one rural oncology clinic in 2013	Australian postcodes	Breast, colorectal, lung, esophageal, breast and ovarian, other	45 45 (100%) 0 (0%)	29 (64.4%)	Not stated	QLACS: a ppearance-related concerns, benefits of cancer, family-related distress, recurrence-related distress, financial problems
[39] Meneses et al. 2009	United States	Randomized controlled trial	Examined a Breast Cancer Education Intervention (BCEI) comprising, firstly, education and support delivered face to face or via telephone and, secondly, written and audio-recorded information on physical, psychological, social, and spiritual well-being	Recruitment through regional cancer center and private oncology offices. Recruitment period not stated	U.S. Census Bureau definition or Florida Index of Treatment Accessibility (FITA) score	Breast	53 53 (100%) 0 (0%)	53 (100%)	≤ 1 (100%) 8.6 months (mean) 2.7 months (SD)	QOL-BCS (2005): p hysical, psychological, social, spiritual, global
[40] Meneses et al. 2020	United States	Randomized controlled trial	Examined a telephone-based education and support intervention comprising self-management of symptoms and late effects, cancer surveillance, health and wellness, and psychosocial well-being for breast cancer survivors	Recruitment through Florida Cancer Registry. Recruitment period not stated	2000 Florida statute of Index of Research Access (IRA)	Breast	432 432 (100%) 0 (0%)	432 (100%)	25.6 months (mean) 7.9 months (SD)	QOL-BCS (2008): Global SF-36: p hysical, mental
[41] Modesitt et al. 2020	United States	Prospective, pre-post	Assessed the outcomes of an exercise intervention with motivational coaching on cancer survivors who were overweight or obese	Recruitment through a cancer center. Recruitment period not stated but ethics approval obtained in 2015	No specific classification reported	Breast, endometrial, ovarian	99 99 (100%) 0 (0%)	99 (100%)	Not stated	SF-36v2: p hysical, mental
[42] Pedro et al. 2014^]^	United States	Cross-sectional	Examined demographic and clinical correlates of HRQOL and levels of rurality in breast cancer survivors	Recruitment through Colorado Central Cancer Registry. Recruitment period not stated	Rural/Urban Continuum Codes (RUCC)	Breast, colon, hematological, prostate, other	91 91 (100%) 0 (0%)	59 (64.8%)	5–10 (45%) 11–20 (45%) > 20 (2%) Not stated (3%)	EORTC QLQ-C30: p hysical, emotional, social, role, cognitive, global
[44] Santoyo-Olsson et al. 2023	United States	Cross-sectional	Used data from the Nuevo Amanecer-II Randomised Controlled Trial, which assessed associations between limited English language proficiency, engagement with physicians, and HRQOL among Latino breast cancer survivors	Recruitment through community cancer centers and hospitals between 2011–2014 and 2016–2018	U.S. Census data and maps to identify geographically defined counties of California: rural defined as counties whose economy is heavily based on agriculture	Breast	304 153 (50.3%) 151 (49.7%)	304 (100%)	Not stated	FACT-G: p hysical, emotional, social/family, functional, total score
[50] Socha et al. 2021	Poland	Cross-sectional	Examined demographic and clinical correlates of HRQOL among breast cancer survivors in Poland	Recruitment through consumer self-help organization. Recruitment period not stated	No specific classification reported	Breast	250 24 (9.6%) 226 (90.4%)	250 (100%)	9.4 (mean) 6.5 (SD)	SF-36: p hysical, mental
[43] Strayhorn et al. 2020	United States	Cross-sectional	Examined the impact of physical and psychological comorbidities and treatment-related symptoms on HRQOL in rural cancer survivors	Recruitment through flyers, list servers, and social media in cancer centers, public health departments, clinics, hospitals, churches, hair salons, support groups, cancer-related events, and word-of-mouth. Commercial list of landline and cellular phone numbers. Recruitment between Jan 2017 and Feb 2018 and Mar 2018 and Sept 2018	Rural/Urban Continuum Codes (RUCC)	Breast, gastrointestinal, gynecological, lymphoma, skin, other	125 125 (100%) 0 (0%)	102 (81.6%)	Not stated	FACT-G: s ocial/family, functional SF-12: physical, mental
[46] Thomas et al. 2014	Ireland	Cross-sectional	Examined demographic and clinical correlates of HRQOL in head and neck cancer survivors	Recruited through the National Cancer Registry Ireland in 2012	Composite measure: settlement size, population density, and proximity to the patient’s treatment center	Head and neck	575 214 (37.2%) 361 (62.8%)	188 (32.7%)	> 8 months (100%)	FACT-G (with FACT-HN): p hysical, emotional, social/family, functional, head and neck cancer well-being
[48] Vallance et al. 2012	Canada	Cross-sectional	Examined the impact of fatigue and physical activity levels on HRQOL in rural breast cancer survivors	Recruited through Alberta Cancer Registry between September and October 2009	Canada’s Census of Population	Breast	524 524 (100%) 0 (0%)	524 (100%)	76.4 months (mean)	FACT-G: p hysical, emotional, social/family, functional, total score

^†^The same study. *EORTC-QLQ-C30* European Organisation for Research and Treatment of Cancer Quality of Life Questionnaire, *FACT-G* Functional Assessment of Cancer Therapy-General, *FACT-B* Functional Assessment of Cancer Therapy-Breast, *FACT-B (Spanish)* Functional Assessment of Cancer Therapy-Breast (Spanish), *FACT-B* + *4* Functional Assessment of Cancer Therapy-Lymphedema, *FACT-ES* Functional Assessment of Cancer Therapy-Endocrine Symptoms, *FACT-HN* Functional Assessment of Cancer Therapy-Head and Neck, *HRQOL* health-related quality of life, *QLACS* Quality of Life in Adult Cancer Survivors tool, *QOL-BCS* Quality of Life Breast Cancer questionnaire, *QOL*-*Breast Cancer* quality of life–breast cancer, *SF-12* Medical Outcomes Study Short-Form-12, *SF-36* Medical Outcomes Short-Form 36, *SF-36v2* Medical Outcomes Short-Form 36 (version 2), *SD* standard deviation

Across the 15 studies, there were 5579 participants; 58% were rural-residing. The mean of mean ages reported across 11 studies was 60.3 (standard deviation (SD) = 6.2) years. The number of years since diagnosis was reported in 9 studies (4580 participants), with 27% ≤ 1-year post-diagnosis, 44% between 1 and 5 years, and 30% more than 5. Nine studies (10 papers) were conducted among breast cancer survivors alone [31, 36, 37, 39, 40, 44, 47–50] and comprised 80% of the total participant sample. Eight studies (nine papers) reported rural-only data [36, 37, 39–43, 45, 48], and seven studies (seven papers) provided a comparison with an urban population [31, 38, 44, 46, 47, 49, 50].

The most commonly used HRQOL instruments were from the Functional Assessment of Chronic Illness Therapy (FACIT) suite [31, 36, 43, 44, 46–48, 51] and SF-12/SF-36 [37, 38, 40, 41, 43, 50]. Three studies used instruments specific to the post-treatment cancer survivorship phase: Quality of Life in Adult Cancer Survivors (QLACS), Quality of Life–Breast Cancer Survivors (QOL-BCS), and Quality of Life–Breast Cancer (QOL-Breast Cancer) [39, 45, 49]. For most instruments, a higher score represented better HRQOL; however, on the QOL-BCS, a lower score represented better HRQOL. On the QLACS, a higher score in the “benefit” domain indicated better HRQOL, but a higher score on the “appearance,” “family,” “recurrence,” and “financial” domains indicated worse HRQOL.

Normative data were available for the FACT-G [30, 31], SF-12/-36 [32], and EORTC QLQ-C30 [33] instruments. MCID was available for the FACT-G [30] and SF-12/-36 [34], while TCI data was available for the EORTC QLQ-C30 [35]. No normative or clinically important data could be located for the QLACS, QOL-BCS, or QOL-Breast Cancer instruments.

### Quality assessment

All 11 cross-sectional studies were of high quality [31, 36, 42–50]. The three randomized controlled trials (RCT) were of moderate quality, largely due to lack of clearly documented methodologies relating to randomization and blinding [38–40]. However, given only baseline HRQOL data were extracted, and this was well-documented, it is unclear whether or not these methodological issues would have had any bearing on extracted HRQOL data. Of the two studies assessed against the pre- and post-intervention checklist, one was rated as high quality [37] and the other as moderate quality [41] (Supplementary Material 2).

### HRQOL in rural cancer survivors

To avoid duplication of data, data from the DiSipio et al. [31] paper is not reported in the following sections. All study means, normative means, and clinically important values are detailed in Supplementary Material 3.

### Global HRQOL

Normative data for global HRQOL were available for comparison for four studies [36, 42, 44, 48] and MCID values for three studies [36, 44, 48]. Of these studies, global HRQOL in RCS was statistically and clinically better than normative populations in one study with breast cancer survivors in Canada [48] and worse in one study with Spanish speaking Latina breast cancer survivors in the US [44]. In one study with Australian breast cancer survivors [36], there were no statistical or clinically significant differences between study and normative populations (Table 2).

**Table 2:**
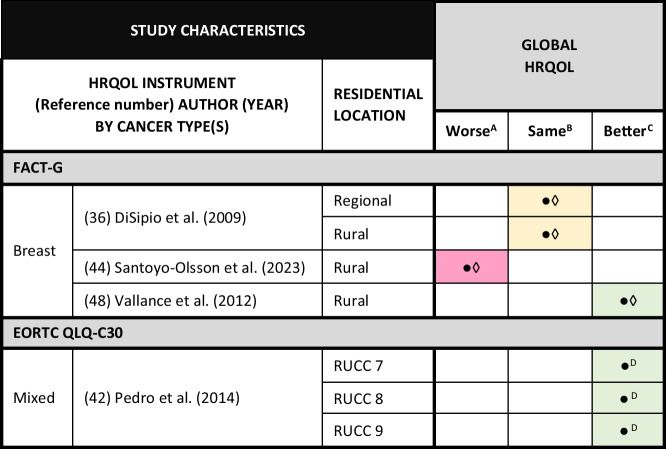
Rural cancer survivors’ global health-related quality of life (HRQOL) compared with normative and clinically significant values

● Statistical significance: study mean score compared with normative mean score

◊ Clinical significance: study mean score compared with minimum clinically important difference or threshold for clinical importance values

^A^Statistically significant difference between study and normative mean; i.e., no cross-over in study and normative 95% confidence intervals. HRQOL is clinically worse in the study population than the normative population (pink shading)

^B^No statistically significant difference between study and normative mean; i.e., cross-over in study and normative 95% confidence intervals. No clinical difference in HRQOL between study population and normative population (yellow shading)

^C^Statistically significant difference between study and normative mean; i.e., no cross-over in study and normative 95% confidence intervals. HRQOL is clinically better in the study population than the normative population (green shading)

^D^Global threshold for clinical importance values not available for the EORTC QLQ-C30 instrument

*EORTC QLQ-C30* European Organisation for Research and Treatment of Cancer Quality of Life Core-30; *FACT-G*: Functional Assessment of Cancer Therapy-General;*RUCC* Rural–Urban Continuum Codes; RUCC 7 nonmetro county with an urban population of 2500–19,999, not adjacent to a metro area; *RUCC 8* nonmetro county completely rural or less than 2500 urban population, adjacent to metro area; *RUCC 9* nonmetro county completely rural or less than 2500 urban population, not adjacent to metro area

### Domain-specific HRQOL

Normative data and MCID and TCI values were available for comparison for 12 studies with physical and emotional or/mental HRQOL [36–38, 40–44, 46–48, 50]. Physical HRQOL in RCS was statistically and clinically worse than normative populations in 5/12 studies [36, 38, 40, 43, 44] and better in 1/12 studies [42]. Emotional or/mental HRQOL was statistically and clinically worse than normative populations in 1/12 studies [44] and better in 2/12 studies [38, 42]. In many studies (7/12), there were no differences between in emotional or/mental HRQOL between study participants and normative populations [40, 41, 43, 46–48, 50] (Table 3).

**Table 3:**
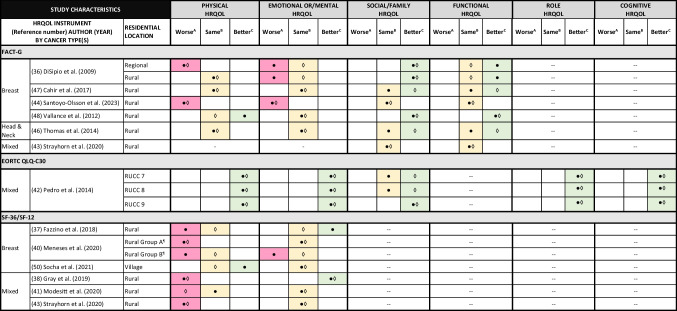
Rural cancer survivors’ domain-specific health-related quality of life (HRQOL) compared with normative and clinically significant values

● Statistical significance: study mean score compared with normative mean score

◊ Clinical significance: study mean score compared with minimum clinically important difference or threshold for clinical importance values

- HRQOL values not reported in article

-- HRQOL domain not collected in instrument

Of the seven studies with normative data and MCID values for social/family HRQOL, RCS had clinically better social/family than normative populations in 5/7 studies [36, 42, 46–48]. There were no studies with worse social/family HRQOL in RCS compared with normative populations (Table 3).

Of the six studies with normative data and MCID values for functional HRQOL, RCS had either clinically better functional HRQOL (3/6 studies) [46–48], or there were no differences compared with normative populations (3/6 studies) [36, 43, 44]. There were no studies with worse functional HRQOL in RCS compared with normative populations (Table 3).

Role and cognitive HRQOL was reported in one study [42], with statistically and clinically better role and cognitive HRQOL in RCS than normative populations (Table 3).

### Studies without normative and MCID/TCI data

There were four studies where normative and MCID/TCI data were unavailable [39, 40, 45, 49] (Supplementary Material 4). Of these, three studies were with breast cancer survivors, two were in the US using the QOL-BCS [39, 40], and one was in Pakistan using the QOL-Breast Cancer instrument [49]. The US-based studies showed high mean global or/total, physical, psychological, social, and spiritual scores, while the Pakistan-based study showed low mean scores across all these domains. One study reported on the QLACS among a mix of cancer types in 45 Australian RCS [45]. This study reported high mean scores in the “benefits of cancer” domain and low mean scores in “appearance-related concerns,” “family-related distress,” “recurrence-related distress,” and “financial stress” domains.

### HRQOL in RCS compared to their UCS counterparts

Seven studies examined HRQOL differences between RCS and UCS [31, 38, 44, 46, 47, 49, 50]; multivariable analysis, normative data and MCID values were available for six studies [31, 38, 44, 46, 47, 50] (Table 4 and Supplementary Material 5).

**Table 4:**
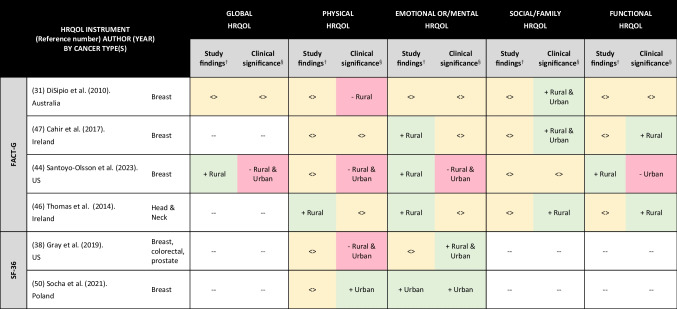
Health-related quality of life (HRQOL) between rural and urban cancer survivors, multivariable analysis results, and clinical significance

^†^Results based on multivariable analysis as reported in each paper

^§^Clinically significant compared with minimum clinically important difference values

<  > , no difference in health-related quality of life between rural and urban populations (yellow shading)

-, worse health-related quality of life (pink shading)

+ , better health-related quality of life (green shading)

--, not collected/reported/relevant to instrument

*FACT- G* Functional Assessment of Cancer Therapy-General. HRQOL; *SF-36* Medical Outcomes Study Short-Form 36

Global HRQOL was reported in two studies: one found no differences between RCS and UCS as well as no differences from normative populations [31], while the other reported better global HRQOL in RCS than UCS even though both RCS and UCS had worse global HRQOL than normative populations [44].

Many studies reported no differences in multivariable analysis between RCS and UCS in physical (5/6 studies) [31, 38, 44, 47, 50], social/family (4/4 studies) [36, 44, 46, 47], and functional (3/4 studies) [36, 46, 47] HRQOL. However, when we compared mean study scores with mean normative population scores, we found RCS and UCS had clinically worse physical HRQOL in 3/6 studies [31, 38, 44] and 2/6 studies [38, 44] respectively. We also found RCS and UCS had clinically better social/family HRQOL than normative populations in 3/4 studies [31, 46, 47] and 2/4 studies [31, 47] respectively. RCS had clinically better functional HRQOL than normative populations in 2/4 studies [46, 47], while UCS had clinically worse functional HRQOL than normative populations in 1/4 studies [44].

In multivariable analysis of emotional or/mental HRQOL, RCS had better emotional or/mental HRQOL than UCS in 3/6 studies [44, 46, 47], while UCS had better emotional or/mental HRQOL than RCS in 1/6 studies [50]. When we compared mean study scores with mean normative population scores, we found that 3/6 studies [31, 46, 47] showed no clinical differences in emotional or/mental HRQOL between normative populations and each of RCS and UCS. One of six studies [44] showed worse emotional or/mental HRQOL in RCS and UCS compared with normative data, while a different study [38] showed better emotional or/mental HRQOL in RCS and UCS compared with normative data. In another of the six studies [50], there was better emotional or/mental HRQOL in UCS relative to normative population data.

### Rural–urban studies without multivariable, normative, or clinical data

One study used the QOL-Breast Cancer instrument and reported on a sample of 250 breast cancer survivors in Pakistan [49]. On univariable analysis, this study reported UCS had statistically significantly better global or/total and spiritual HRQOL than RCS, and RCS had better physical and emotional HRQOL than UCS. There were no differences between cohorts in social HRQOL. While these analyses showed differences in HRQOL between the two groups, as described above, the mean scores in this study were at the low end of the scoring range, indicating generally worse HRQOL in both cohorts (Supplementary Material 6).

Three studies reported on tumor-specific domains [31, 46, 47]. One Australian study found RCS had statistically significantly lower scores on the breast well-being subscale of the FACT-B + 4 than UCS [31]. Another found Irish RCS had a statistically significantly lower endocrine symptom burden on the FACT-ES subscale than UCS [47]. The third study found that Irish RCS experienced statistically significantly fewer head and neck cancer–specific concerns on the FACT-HN than their urban counterparts [46] (Supplementary material 6).

## Discussion

Our study presents the first comprehensive review of HRQOL in RCS and makes novel comparisons with UCS and normative populations. We found that many studies did not indicate worse HRQOL in RCS, and in domains such as social/family, functional, and emotional/mental, HRQOL was equal or better in RCS than UCS and/or normative populations. The review also illustrates the need to benchmark HRQOL against normative and clinically important values, as the HRQOL differences between rural–urban populations reported in some studies were not clinically significant compared with normative and clinically important values.

Drawing conclusions about global HRQOL from this review was limited to very few studies conducted mainly with breast cancer survivors [31, 36, 42, 44, 48]. Only one study showed worse global/total HRQOL in RCS compared with UCS and normative populations [44]. This study was conducted in the US with a culturally and linguistically diverse (CALD) population (Spanish-speaking Latina people), 85% of whom had no/low English language proficiency. Comparing the impact of CALD status on HRQOL with other studies in our review is also limited, as only two other studies were conducted in mainly non-English-speaking countries (Pakistan [49] and Poland [50]), and global or normative data were unavailable for these studies. Furthermore, as HRQOL is a multidimensional construct, using an aggregated score may mask potential differences in specific HRQOL domains and obscure where cancer survivors may require specific assistance [52]. Additionally, instruments such as the SF-12/36 do not calculate a global score and are excluded from comparisons. It may be more meaningful to examine domain-specific HRQOL, with a view to identifying which aspects of HRQOL might be affected and determining which areas of follow-up care to prioritize.

We can make inferences about physical and emotional/mental HRQOL as these were the most reported HRQOL domains. Our findings of poor physical HRQOL in RCS and UCS are consistent with other studies showing the persistence of physical problems post-treatment [4, 5]. This suggests a need for services which support cancer survivors to manage long-term impacts and late-effects of cancer and its treatment. While an ever-growing number of studies highlight the benefits of physical activity interventions for cancer survivors [53, 54], the availability of such interventions are limited in rural areas [55]. Although telephone, video and online learning programs can increase access, these delivery modes are not always effective in meeting the physical needs of RCS [56]. Place-based programs that reflect the sociodemographic and access needs of RCS are required.

In our review, emotional/mental HRQOL in RCS and UCS was found to be generally similar to normative populations. This finding also supports a systematic review which found no differences in psychosocial morbidity and unmet needs between urban and rural cancer patients/survivors [57]. It has also been hypothesized that better emotional well-being in RCS may relate to personal characteristics attributed to individuals who choose to live in rural areas, including being inherently more resilient and self-sufficient [42]. Increased resilience has been shown to be associated with fewer self-reported unmet needs [58], which may be a marker of HRQOL. Research has also identified RCS’ increased stoicism and lower expectations of their health service as contributing factors [59]. Whether these psychological traits are also characteristic of UCS needs to be explored.

Our review found that RCS and UCS had generally better social/family HRQOL than normative populations, and RCS had better functional, role, and cognitive HRQOL than UCS and normative populations. It has been hypothesized that rural communities have stronger social networks and are more “tight-knit”, contributing to improved HRQOL [42, 51]. It has also been suggested that in urban settings, greater access to and availability of support services, networks and resources may improve social HRQOL [60, 61]. These findings should be considered alongside the fact that not all instruments collect these domains, and instruments such as the SF12/36 incorporate a social subdomain as part of the mental HRQOL composite score. Furthermore, of the three studies with better social HRQOL in RCS and UCS relative to normative populations [31, 46, 47], two were from one country (Ireland) [46, 47] and two with breast cancer survivors [31, 47]. Hence, further research is required to build on these findings.

Only three HRQOL instruments were specific to the survivorship setting (QLACS, QOL-BCS and QOL-Breast Cancer), with two specifically for breast cancer survivors and none with normative or clinical importance data for comparison. While FACT instruments have been validated in cancer survivor samples [62], they and other instruments reported in this review are primarily intended for the treatment phase and do not capture data on common survivorship issues. Furthermore, spiritual well-being was only examined in two studies with breast cancer survivors [39, 49]. Positive spiritual well-being has been associated with improvements in physical and emotional HRQOL in cancer survivors broadly [63, 64].

Two studies investigated the impact of remoteness on HRQOL [36, 42], with one study indicating higher HRQOL in RCS in the most remote region (RUCC 9) compared with other regional areas (RUCC 7 and 8) and normative populations [42]. Further studies are needed to understand whether HRQOL varies by remoteness and to identify factors that may contribute to any such variation. Distance and travel are only one dimension of access. Russell et al. [65] argue that dimensions such as availability, geography, affordability, accommodation, timeliness, acceptability, and awareness are important when assessing access to healthcare services for rural residents. Furthermore, definitions of, and criteria for, remoteness vary between countries and geographical (spatial) distance alone may be too crude an indicator of place-based disadvantage.

This review has limitations. Our definition of cancer survivor excludes people with advanced/metastatic disease who may live for years. There is also no universal HRQOL definition. Despite using broad search terms, we may have missed some relevant papers. Caution is also needed when comparing domains across instruments; while the constructs of HRQOL were similar across instruments, the questions asked and the way they were scored differ between instruments. A focus on papers published in English may have also excluded papers from non-English-speaking countries and authors.

## Conclusion

It is currently impossible to definitively determine if HRQOL varies between RCS and UCS due to the limited primary literature designed to assess this. Further research using survivorship-specific HRQOL instruments is required to identify issues of importance in the survivorship phase, as well as a diversity of cancer types and rural populations. Future studies including RCS would help inform the development of tailored interventions and uniquely rural approaches to assessment and management of cancer in the survivorship setting.

### Supplementary Information

Below is the link to the electronic supplementary material.

Supplementary file1 (DOCX 80 KB)

## Data Availability

Supplementary tables provided.
