# Supplementary material for: Health-related quality of life in rural cancer survivors compared with their urban counterparts: a systematic review
Source: Support Care Cancer. 2024 Jun 12;32(7):424. doi: 10.1007/s00520-024-08618-9 (PMC11168981; doi:10.1007/s00520-024-08618-9)
Supplement: Supplementary file 1 — Supplementary file1 (DOCX 80 KB) [file 520_2024_8618_MOESM1_ESM.docx]

**Supplementary Material 1: Ovid Medline Example Search**

| Set | Search Statement |
| --- | --- |
| 1. | Rural*.mp. [mp=title, abstract, original title, name of substance word, subject heading word, floating sub-heading word, keyword heading word, organism supplementary concept word, protocol supplementary concept word, rare disease supplementary concept word, unique identifier, synonyms] |
| 2. | regional*.mp. [mp=title, abstract, original title, name of substance word, subject heading word, floating sub-heading word, keyword heading word, organism supplementary concept word, protocol supplementary concept word, rare disease supplementary concept word, unique identifier, synonyms] |
| 3. | remote*.mp. [mp=title, abstract, original title, name of substance word, subject heading word, floating sub-heading word, keyword heading word, organism supplementary concept word, protocol supplementary concept word, rare disease supplementary concept word, unique identifier, synonyms] |
| 4. | non urban.mp. [mp=title, abstract, original title, name of substance word, subject heading word, floating sub-heading word, keyword heading word, organism supplementary concept word, protocol supplementary concept word, rare disease supplementary concept word, unique identifier, synonyms] |
| 5. | non metropolitan.mp. [mp=title, abstract, original title, name of substance word, subject heading word, floating sub-heading word, keyword heading word, organism supplementary concept word, protocol supplementary concept word, rare disease supplementary concept word, unique identifier, synonyms] |
| 6. | urban.mp. [mp=title, abstract, original title, name of substance word, subject heading word, floating sub-heading word, keyword heading word, organism supplementary concept word, protocol supplementary concept word, rare disease supplementary concept word, unique identifier, synonyms] |
| 7. | metro*.mp. [mp=title, abstract, original title, name of substance word, subject heading word, floating sub-heading word, keyword heading word, organism supplementary concept word, protocol supplementary concept word, rare disease supplementary concept word, unique identifier, synonyms] |
| 8. | non rural.mp. [mp=title, abstract, original title, name of substance word, subject heading word, floating sub-heading word, keyword heading word, organism supplementary concept word, protocol supplementary concept word, rare disease supplementary concept word, unique identifier, synonyms] |
| 9. | 1 or 2 or 3 or 4 or 5 |
| 10. | 6 or 7 or 8 |
| 11. | 9 or 10 |
| 12. | exp Neoplasms/ |
| 13. | cancer*.mp. [mp=title, abstract, original title, name of substance word, subject heading word, floating sub-heading word, keyword heading word, organism supplementary concept word, protocol supplementary concept word, rare disease supplementary concept word, unique identifier, synonyms] |
| 14. | 12 or 13 |
| 15. | survivors/ or exp cancer survivors/ |
| 16. | survivor*.mp. [mp=title, abstract, original title, name of substance word, subject heading word, floating sub-heading word, keyword heading word, organism supplementary concept word, protocol supplementary concept word, rare disease supplementary concept word, unique identifier, synonyms] |
| 17. | post-treatment.mp. [mp=title, abstract, original title, name of substance word, subject heading word, floating sub-heading word, keyword heading word, organism supplementary concept word, protocol supplementary concept word, rare disease supplementary concept word, unique identifier, synonyms] |
| 18. | 15 or 16 or 17 |
| 19. | quality of life.mp. [mp=title, abstract, original title, name of substance word, subject heading word, floating sub-heading word, keyword heading word, organism supplementary concept word, protocol supplementary concept word, rare disease supplementary concept word, unique identifier, synonyms] |
| 20. | well-being.mp. [mp=title, abstract, original title, name of substance word, subject heading word, floating sub-heading word, keyword heading word, organism supplementary concept word, protocol supplementary concept word, rare disease supplementary concept word, unique identifier, synonyms] |
| 21. | wellbeing.mp. [mp=title, abstract, original title, name of substance word, subject heading word, floating sub-heading word, keyword heading word, organism supplementary concept word, protocol supplementary concept word, rare disease supplementary concept word, unique identifier, synonyms] |
| 22. | life satisfaction.mp. [mp=title, abstract, original title, name of substance word, subject heading word, floating sub-heading word, keyword heading word, organism supplementary concept word, protocol supplementary concept word, rare disease supplementary concept word, unique identifier, synonyms] |
| 23. | 19 or 20 or 21 or 22 |
| 24. | 11 and 14 and 18 and 23 |
| 25. | limit 24 to (english language and yr="2021 -Current") |

**Supplementary material 2: JBI assessment**

**Table 2a: Studies assessed using the JBI Checklist for Analytic Cross-Sectional Studies (26)**

| **Checklist questions** | **Azam et al. 2021 (49)** | **Cahir et al. 2017 (47)** | **Disipio et al. 2010 (31)** | **DiSipio et al. 2009 (36)** | **Mandaliya et al. 2016 (45)** | **Pedro et al. 2014 (42)** | **Santoyo-Olsson et al. 2023 (44)** | **Socha et al. 2021 (50)** | **Strayhorn et al. 2020 (43)** | **Thomas et al. 2014 (46)** | **Vallance et al. 2012 (48)** |
| --- | --- | --- | --- | --- | --- | --- | --- | --- | --- | --- | --- |
| Were the criteria for inclusion in the sample clearly defined? | Yes | Yes | Yes | Yes | Yes | Yes | Yes | Yes | Yes | Yes | Yes |
| Were the study subjects and the setting described in detail? | Unclear | Yes | Yes | Yes | Yes | Yes | Yes | Yes | Yes | Yes | Yes |
| Was the exposure measured in a valid and reliable way? | Yes | Yes | Yes | Yes | Yes | Yes | Yes | Yes | Yes | Yes | Yes |
| Were objective, standard criteria used for the measurement of the condition? | Unclear | Yes | Unclear | Yes | Unclear | Unclear | Unclear | Unclear | Unclear | Yes | Yes |
| Were there confounding factors identified? | Yes | Yes | Yes | Yes | Yes | Yes | Yes | Yes | Yes | Yes | Yes |
| Were strategies to deal with confounding factors stated? | Yes | Yes | Yes | Yes | Yes | Yes | Yes | Yes | Yes | Yes | Yes |
| Were the outcomes measured in a valid and reliable way? | Yes | Yes | Yes | Yes | Yes | Yes | Yes | Yes | Yes | Yes | Yes |
| Was appropriate statistical analysis used? | Yes | Yes | Yes | Yes | Yes | Yes | Yes | Yes | Yes | Yes | Yes |
| Overall appraisal category: | Include | Include | Include | Include | Include | Include | Include | Include | Include | Include | Include |
| Quality Assessment: | 6/8 - High | 8/8 - High | 7/8 - High | 8/8 - High | 7/8 - High | 7/8 - High | 7/8 - High | 7/8 - High | 7/8 - High | 8/8 – High | 8/8 - High |

**Table 2b: Studies assessed using the JBI Checklist for Randomized Controlled Trials (25)**

| **Checklist questions** | **Gray et al. 2019 (38)** | **Meneses et al. 2009 (39)** | **Meneses et al. 2020 (40)** |
| --- | --- | --- | --- |
| Was true randomisation used for assignment of participants to treatment groups? | Yes | Yes | Unclear |
| Was allocation to treatment groups concealed? | No | No | No |
| Were treatment groups similar at the baseline? | Yes | Yes | Yes |
| Were participants blind to treatment assignment? | No | No | No |
| Were those delivering treatment blind to treatment assignment? | Unclear | Unclear | Unclear |
| Were outcomes assessors blind to treatment assignment? | Unclear | Unclear | Unclear |
| Were treatment groups treated identically other than the intervention of interest? | N/A | N/A | N/A |
| Was follow up complete and if not, were differences between groups in terms of their follow up adequately described and analysed? | N/A | N/A | N/A |
| Were participants analysed in the groups to which they were randomised? | No | Yes | Yes |
| Were outcomes measured in the same way for treatment groups? | Yes | Yes | Yes |
| Were outcomes measured in a reliable way? | Yes | Yes | Yes |
| Was appropriate statistical analysis used? | Yes | Yes | Yes |
| Was the trial design appropriate and any deviations from the standard RCT design (individual randomisation, parallel groups) accounted for in the conduct and analysis of the trial? | N/A | N/A | N/A |
| Overall appraisal category: | Include | Include | Include |
| Quality Assessment: | 5/10 - Moderate | 6/10 - Moderate | 5/10 - Moderate |

**Table 2c: Studies assessed using the JBI Checklist for Quasi-experimental studies (26)**

| **Checklist questions** | **Fazzino et al. 2018 (37)** | **Modesitt et al. 2016 (41)** |
| --- | --- | --- |
| Is it clear in the study what is the 'cause' and what is the 'effect' (i.e. there is no confusion about which variable comes first)? | Yes | Yes |
| Were the participants included in any comparisons similar? | Yes | Yes |
| Were the participants included in any comparisons receiving similar treatment/care, other than the exposure or intervention of interest? | Yes | Yes |
| Was there a control group? | No | No |
| Were there multiple measurements of the outcome both pre and post the intervention/exposure? | Yes | No |
| Was follow up complete and if not, were differences between groups I terms of their follow up adequately described and analysed? | Yes | No |
| Were the outcomes of participants included in any comparisons measured in the same way? | Yes | Yes |
| Were outcomes measured in a reliable way? | Yes | Yes |
| Was appropriate statistical analysis used? | Yes | Yes |
| Overall appraisal category: | Include | Include |
| Quality Assessment: | 8/9 - High | 6/9 - Moderate |

**Supplementary material 3: Study means, normative means and clinically important values**

**Table 3a: Rural cancer survivors’ global health-related quality of life (HRQOL) compared with normative and clinically significant values (n=4 studies)**

| **STUDY CHARACTERISTICS** | | | **GLOBAL HEALTH-RELATED QUALITY OF LIFE** | | | | |
| --- | --- | --- | --- | --- | --- | --- | --- |
|  |  |  | **STATISTICAL SIGNIFICANCE** | | **CLINICAL IMPORTANCE** | | |
| **HRQOL INSTRUMENT (SCORING RANGE)**  **(Reference number)** **AUTHOR (YEAR)**  **BY CANCER TYPE(S)** | | **RESIDENTIAL LOCATION** | **STUDY**  **MEAN (95% CI)** | **NORMATIVE**  **MEAN (95% CI)** | **DIFFERENCE (STUDY MEAN -**  **NORMATIVE MEAN)** | **ANCHORS** | **DIRECTION**^§^ |
| **FACT-G (0-108)** | | | **p<0.05** | **p<0.05** |  | **≥5 MCID POINTS**^◊^ |  |
| Breast | (36) DiSipio et al. (2009) | Regional | 86.1 (83.9, 88.3) | 85.8 (84.4, 87.3) | 0.3 | X | <> |
|  |  | Rural | 87.6 (84.7, 90.4) | 85.8 (84.4, 87.3) | 1.8 | X | <> |
|  | (44) Santoyo-Olsson et al. (2023) | Rural | 74.0 (71.5, 76.5) | 80.1 (78.6, 81.6)* | -6.1 | 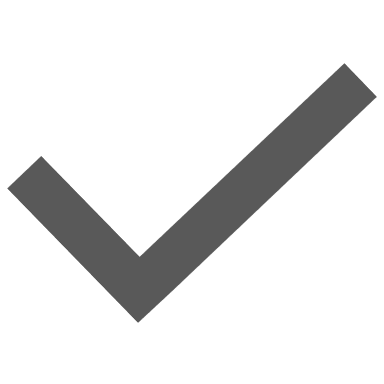 | - |
|  | (48) Vallance et al. (2012) | Rural | 88.6 (87.3, 89.9) | 80.1 (78.6, 81.6)* | 8.5 | 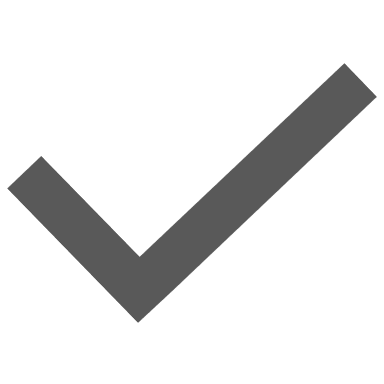 | + |
| **EORTC QLQ-C30 (0-100)** | | | **p<0.05** | **p<0.05** |  | **TCI (NA)** |  |
| Mixed | (42) Pedro et al. (2014) | RUCC 7 | 78.2 (73.0, 83.5) | 61.3 (61.0, 61.6)* | 16.9 | NA | NA |
|  |  | RUCC 8 | 79.8 (73.7, 85.8) | 61.3 (61.0, 61.6)* | 18.5 | NA | NA |
|  |  | RUCC 9 | 83.3 (77.2, 89.5) | 61.3 (61.0, 61.6)* | 22.0 | NA | NA |

* Statistically significant difference between study and normative mean; i.e. no cross-over in study and normative 95% confidence intervals.

^◊^ X Not clinically significant; difference between study mean score and normative mean score is <MCID value.
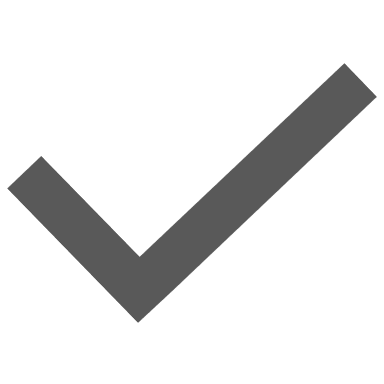
 Clinically significant; difference between study mean score and normative mean score is ≥MCID value.

^§^ <> No clinical difference in HRQOL between study population and normative population (yellow shading). - clinically worse HRQOL in the study population than the normative population (pink shading). + clinically better HRQOL in the study population than the normative population (green shading).

CI – Confidence Interval. European Organisation for Research and Treatment of Cancer Quality of Life Core-30. FACT-G – Functional Assessment of Cancer Therapy – General. MCID – Minimum Clinically Important Difference. NA – global TCI anchor values not available for the EORTC QLQ-C30 instrument. TCI – Threshold for Clinical Importance. RUCC – Rural-Urban Continuum Codes: RUCC 7 Nonmetro county with urban population of 2,500-19,999, not adjacent to a metro area. RUCC 8 Nonmetro county completely rural or less than 2,500 urban population, adjacent. to metro area. RUCC 9 Nonmetro county completely rural or less than 2,500 urban population, not adjacent to metro area

**Table 3b: Rural cancer survivors’ physical health-related quality of life compared with normative and clinically significant values (n=12 studies)**

| **STUDY CHARACTERISTICS** | | | **PHYSICAL HEALTH-RELATED QUALITY OF LIFE** | | | | |
| --- | --- | --- | --- | --- | --- | --- | --- |
|  |  |  | **STATISTICAL SIGNIFICANCE** | | **CLINICAL IMPORTANCE** | | |
| **HRQOL INSTRUMENT (SCORING RANGE)**  **(Reference number)** **AUTHOR (YEAR)**  **BY CANCER TYPE(S)** | | **RESIDENTIAL LOCATION** | **STUDY MEAN (95% CI)** | **NORMATIVE MEAN (95% CI)** | **DIFFERENCE (STUDY MEAN - NORMATIVE MEAN)** | **ANCHORS** | **DIRECTION**^§^ |
| **FACT-G (0-28)** | | | **p<0.05** | **p<0.05** |  | **≥2 MCID POINTS**^◊^ |  |
| Breast | (36) DiSipio et al. (2009) | Regional | 22.7 (22.0, 23.4) | 25.1 (24.7, 25.5)* | -2.4 | 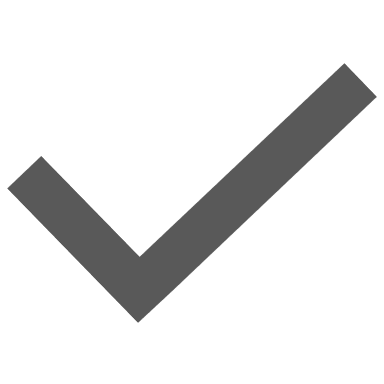 | - |
|  |  | Rural | 23.2 (22.3, 24.1) | 25.1 (24.7, 25.5) | -1.9 | X | <> |
|  | (47) Cahir et al. (2017) | Rural | 25.0 (21.0, 27.0) ^Mdn^ | 24.5 (21.0, 26.8)^Mdn^ | 0.5 | X | <> |
|  | (44) Santoyo-Olsson et al. (2023) | Rural | 20.4 (19.4, 21.4) | 22.7 (22.4, 23.0)* | -2.3 | 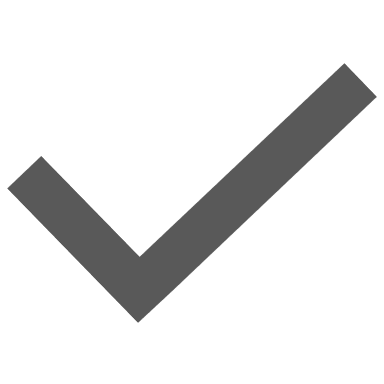 | - |
|  | (48) Vallance et al. (2012) | Rural | 23.7 (23.3, 24.1) | 22.7 (22.4, 23.0)* | 1.0 | X | <> |
| Head & Neck | (46) Thomas et al. (2014) | Rural | 25.0 (21.0, 28.0) ^Mdn^ | 24.5 (21.0, 26.8)^Mdn^ | 0.5 | X | <> |
| **SF-36/SF-12 (0-100)** | | | **p<0.05** | **p<0.05** |  | **≥5 MCID POINTS**^◊^ |  |
| Breast | (37) Fazzino et al. (2018) | Rural | 48.0 (46.8, 49.1) | 50.0 (49.8, 50.2)* | -2.0 | X | <> |
|  | (40) Meneses et al. (2020) | Rural Group A^¶^ | 44.9 (43.5, 46.3) | 50.0 (49.8, 50.2)* | -5.1 | 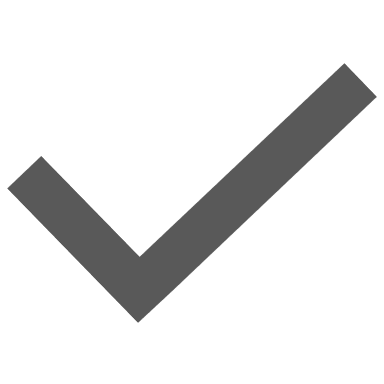 | - |
|  |  | Rural Group B^¶^ | 45.1 (43.7, 46.5) | 50.0 (49.8, 50.2)* | -4.9 | X | <> |
|  | (50) Socha et al. (2021) | Village | 51.7 (44.2, 59.2) | 50.0 (49.8, 50.2)* | 1.7 | X | <> |
| Mixed | (38) Gray et al. (2019) | Rural | 43.7 (42.4, 45.0) | 50.0 (49.8, 50.2)* | -6.3 | 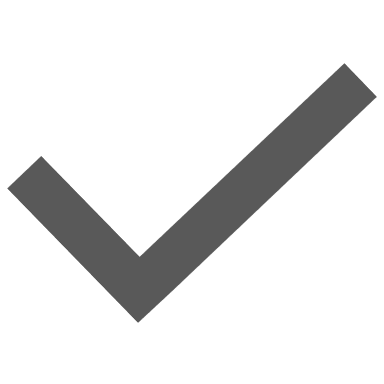 | - |
|  | (41) Modesitt et al. (2020) | Rural | 44.8 (43.2, 46.4)^Mdn^ | 52.6 (42.8, 56.0)^Mdn^ | -7.8 | 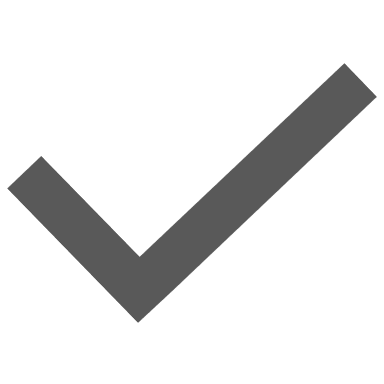 | - |
|  | (43) Strayhorn et al. (2020) | Rural | 45.0 (43.0, 46.9) | 50.0 (49.8, 50.2)* | -5.0 | 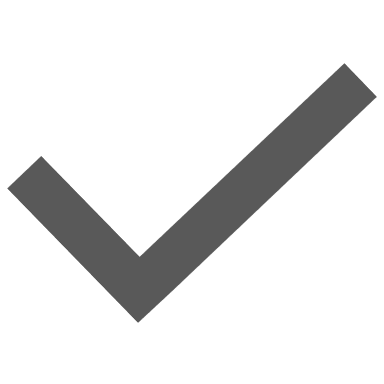 | - |
| **EORTC QLQ-C30 (O-100)** | | | **p<0.05** | **p<0.05** |  | **<83 TCI^□^** |  |
| Mixed | (42) Pedro et al. (2014) | RUCC 7 | 83.5 (77.7, 89.2) | 76.7 (76.4, 77.0)* | 6.8 | X | + |
|  |  | RUCC 8 | 86.4 (80.8, 92.1) | 76.7 (76.4, 77.0)* | 9.7 | X | + |
|  |  | RUCC 9 | 91.0 (86.2, 96.6) | 76.7 (76.4, 77.0)* | 14.3 | X | + |

* Statistically significant difference between study and normative mean; i.e. no cross-over in study and normative 95% confidence intervals.

^◊^ X Not clinically significant; difference between study mean score and normative mean score is <MCID value.
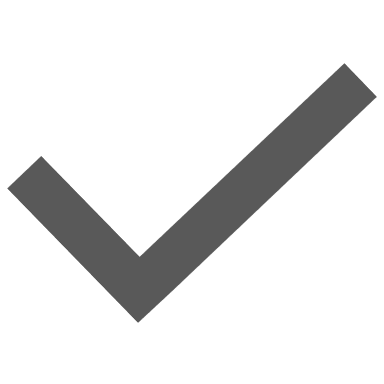
 Clinically significant; difference between study mean score and normative mean score is ≥MCID value.

**^□^** X Not clinically important; the mean study score is above the TCI value.

^§^ <> No clinical difference in HRQOL between study population and normative population (yellow shading). - clinically worse HRQOL in the study population than the normative population (pink shading). + clinically better HRQOL in the study population than the normative population (green shading).

^Mdn^ Median (Interquartile range) instead of mean (95% CI)

^¶^ Rural Group A received early education and support. Rural Group B received Support and delayed education.

CI – Confidence Interval. EORTC QLQ-C30 - European Organisation for Research and Treatment of Cancer Quality of Life Core-30. FACT-G – Functional Assessment of Cancer Therapy – General. MCID – Minimum Clinically Important Difference. TCI – Threshold for Clinical Importance. RUCC – Rural-Urban Continuum Codes: RUCC 7 Nonmetro county with urban population of 2,500-19,999, not adjacent to a metro area. RUCC 8 Nonmetro county completely rural or less than 2,500 urban population, adjacent. to metro area. RUCC 9 Nonmetro county completely rural or less than 2,500 urban population, not adjacent to metro area. SF-36/SF-12- Medical Outcomes Study Short-Form 12 or 36.

**Table 3c: Rural cancer survivors’ emotional/mental health-related quality of life compared with normative and clinically significant values (n=12 studies)**

| **STUDY CHARACTERISTICS** | | | **EMOTIONAL OR/MENTAL HEALTH-RELATED QUALITY OF LIFE** | | | | |
| --- | --- | --- | --- | --- | --- | --- | --- |
|  |  |  | **STATISTICAL SIGNIFICANCE** | | **CLINICAL IMPORTANCE** | | |
| **HRQOL INSTRUMENT (SCORING RANGE)**  **(Reference number)** **AUTHOR (YEAR)**  **BY CANCER TYPE(S)** | | **RESIDENTIAL LOCATION** | **STUDY MEAN (95% CI)** | **NORMATIVE MEAN (95% CI)** | **DIFFERENCE (STUDY MEAN - NORMATIVE MEAN)** | **ANCHORS** | **DIRECTION**^§^ |
| **FACT-G (0-24)** | | | **p<0.05** | **p<0.05** |  | **≥2 MCID POINTS**^◊^ |  |
| Breast | (36) DiSipio et al. (2009) | Regional | 19.4 (18.8, 20.0) | 20.9 (20.5, 21.2)* | -1.5 | X | <> |
|  |  | Rural | 19.2 (18.5, 20.0) | 20.9 (20.5, 21.2)* | -1.7 | X | <> |
|  | (47) Cahir et al. (2017) | Rural | 20.0 (18.0, 23.0)^Mdn^ | 21.0 (18.0, 24.0)^Mdn^ | -1.0 | X | <> |
|  | (44) Santoyo-Olsson et al. (2023) | Rural | 17.3 (16.6, 18.0) | 19.9 (19.6, 20.2)* | -2.6 | 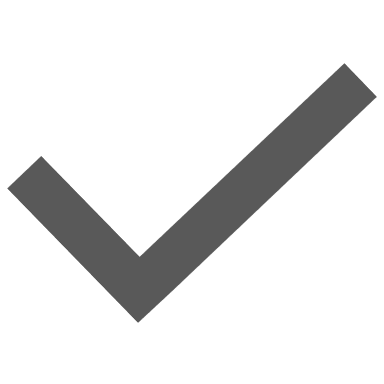 | - |
|  | (48) Vallance et al. (2012) | Rural | 20.0 (19.7, 20.3) | 19.9 (19.6, 20.2) | 0.1 | X | <> |
| Head & Neck | (46) Thomas et al. (2014) | Rural | 21.0 (19.0, 23.0)^Mdn^ | 21.0 (18.0, 24.0)^Mdn^ | 0 | X | <> |
| **SF-36/SF-12 (O-100)** | | | **p<0.05** | **p<0.05** |  | **≥5 MCID POINTS**^◊^ |  |
| Breast | (37) Fazzino et al. (2018) | Rural | 52.4 (51.2, 53.5) | 50.0 (49.8, 50.2)* | 2.4 | X | <> |
|  | (40) Meneses et al. (2020) | Rural Group A^¶^ | 49.5 (48.0, 50.9) | 50.0 (49.8, 50.2) | -0.5 | X | <> |
|  |  | Rural Group B^¶^ | 48.1 (46.5, 49.7) | 50.0 (49.8, 50.2)* | -1.9 | X | <> |
|  | (50) Socha et al. (2021) | Village | 53.9 (44.2, 63.6) | 50.0 (49.8, 50.2) | 3.9 | X | <> |
| Mixed | (38) Gray et al. (2019) | Rural | 56.7 (55.6, 57.8) | 50.0 (49.8, 50.2)* | 6.7 | 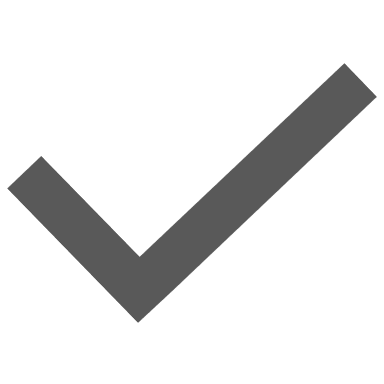 | + |
|  | (41) Modesitt et al. (2020) | Rural | 54.2 (53.0, 55.4)^Mdn^ | 52.5 (45.0, 57.4)^Mdn^ | 1.7 | X | <> |
|  | (43) Strayhorn et al. (2020) | Rural | 48.8 (47.0, 50.7) | 50.0 (49.8, 50.2) | -1.2 | X | <> |
| **EORTC QLQ-C30 (O-100)** | | | **p<0.05** | **p<0.05** |  | **<83 TCI^□^** |  |
| Mixed | (42) Pedro et al. (2014) | RUCC 7 | 81.6 (75.5, 87.7) | 71.4 (71.1, 71.7)* | 10.2 | X | + |
|  |  | RUCC 8 | 82.1 (74.0, 90.3) | 71.4 (71.1, 71.7)* | 10.7 | X | + |
|  |  | RUCC 9 | 88.7 (81.9, 95.5) | 71.4 (71.1, 71.7)* | 17.3 | X | + |

* Statistically significant difference between study and normative mean; i.e. no cross-over in study and normative 95% confidence intervals.

^◊^ X Not clinically significant; difference between study mean score and normative mean score is <MCID value.
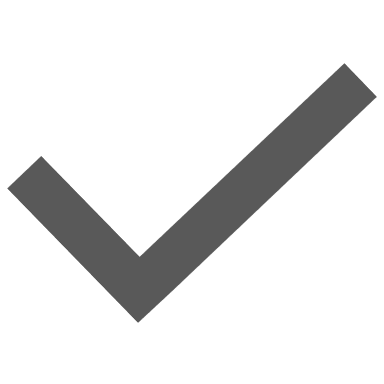
 Clinically significant; difference between study mean score and normative mean score is ≥MCID value.

**^□^** X Not clinically important; the mean study score is above the TCI value.

^§^ <> No clinical difference in HRQOL between study population and normative population (yellow shading). - clinically worse HRQOL in the study population than the normative population (pink shading). + clinically better HRQOL in the study population than the normative population (green shading).

^Mdn^ Median (Interquartile range) instead of mean (95% CI).

^¶^ Rural Group A received early education and support. Rural Group B received Support and delayed education.

CI – Confidence Interval. EORTC QLQ-C30 - European Organisation for Research and Treatment of Cancer Quality of Life Core-30. FACT-G – Functional Assessment of Cancer Therapy – General. MCID – Minimum Clinically Important Difference. TCI – Threshold for Clinical Importance. RUCC – Rural-Urban Continuum Codes: RUCC 7 Nonmetro county with urban population of 2,500-19,999, not adjacent to a metro area. RUCC 8 Nonmetro county completely rural or less than 2,500 urban population, adjacent. to metro area. RUCC 9 Nonmetro county completely rural or less than 2,500 urban population, not adjacent to metro area. SF-36/SF-12- Medical Outcomes Study Short-Form 12 or 36.

**Table 3d: Rural cancer survivors’ social/family health-related quality of life compared with normative and clinically significant values (n=7 studies)**

| **STUDY CHARACTERISTICS** | | | **SOCIAL/FAMILY HEALTH-RELATED QUALITY OF LIFE** | | | | |
| --- | --- | --- | --- | --- | --- | --- | --- |
|  |  |  | **STATISTICAL SIGNIFICANCE** | | **CLINICAL IMPORTANCE** | | |
| **HRQOL INSTRUMENT (SCORING RANGE)**  **(Reference number)** **AUTHOR (YEAR)**  **BY CANCER TYPE(S)** | | **RESIDENTIAL LOCATION** | **STUDY**  **MEAN (95% CI)** | **NORMATIVE**  **MEAN (95% CI)** | **DIFFERENCE (STUDY MEAN - NORMATIVE MEAN)** | **ANCHORS** | **DIRECTION**^§^ |
| **FACT-G (0-28)** | | | **p<0.05** | **p<0.05** |  | **≥2 MCID POINTS**^◊^ |  |
| Breast | (36) DiSipio et al. (2009) | Regional | 22.4 (21.6, 23.2) | 19.6 (19.0, 20.2)* | 2.8 | 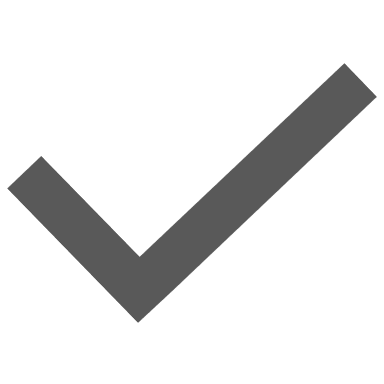 | + |
|  |  | Rural | 22.9 (21.9, 23.9) | 19.6 (19.0, 20.2)* | 3.3 | 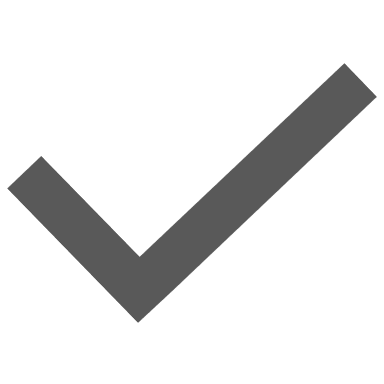 | + |
|  | (47) Cahir et al. (2017) | Rural | 22.0 (17.0, 26.0) ^Mdn^ | 19.6 (14.0, 24.5) ^Mdn^ | 2.4 | 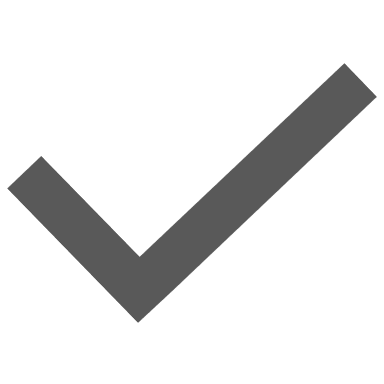 | + |
|  | (44) Santoyo-Olsson et al. (2023) | Rural | 18.2 (17.4, 19.0) | 19.1 (18.7, 19.5) | -0.9 | X | <> |
|  | (48) Vallance et al. (2012) | Rural | 22.2 (21.7, 22.7) | 19.1 (18.7, 19.5)* | 3.1 | 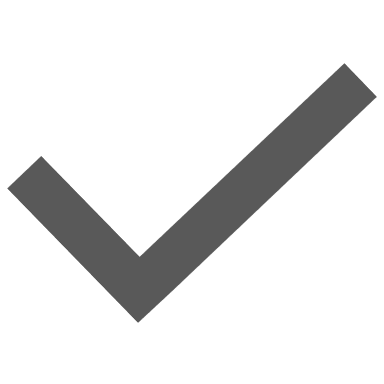 | + |
| Mixed | (43) Strayhorn et al. (2020) | Rural | 18.7 (17.8, 19.6) | 19.1 (18.7, 19.5) | -0.4 | X | <> |
| Head & Neck | (46) Thomas et al. (2014) | Rural | 22.2 (18.0, 25.7) ^Mdn^ | 19.6 (14.0, 24.5) ^Mdn^ | 2.6 | 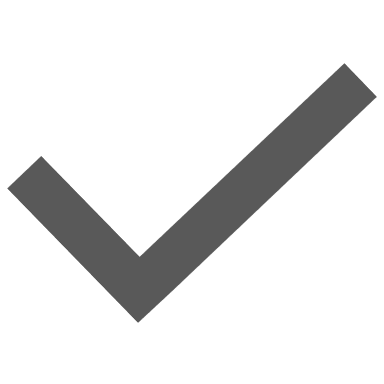 | + |
| **EORTC QLQ-C30 (0-100)** | | | **p<0.05** | **p<0.05** |  | **<58 TCI^□^** |  |
| Mixed | (42) Pedro et al. (2014) | RUCC 7 | 80.6 (72.7, 88.5) | 75 (74.6, 75.4) | 5.6 | X | + |
|  |  | RUCC 8 | 84.5 (74.6, 94.5) | 75 (74.6, 75.4) | 9.5 | X | + |
|  |  | RUCC 9 | 96.4 (92.7, 100.1) | 75 (74.6, 75.4)* | 21.4 | X | + |

* Statistically significant difference between study and normative mean; i.e. no cross-over in study and normative 95% confidence intervals.

^◊^ X Not clinically significant; difference between study mean score and normative mean score is <MCID value.
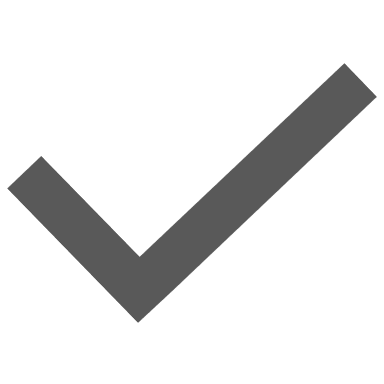
 Clinically significant; difference between study mean score and normative mean score is ≥MCID value.

**^□^** X Not clinically important; the mean study score is above the TCI value.

^§^ <> No clinical difference in HRQOL between study population and normative population (yellow shading). + clinically better HRQOL in the study population than the normative population (green shading).

^Mdn^ Median (Interquartile range) instead of mean (95% CI).

CI – Confidence Interval. EORTC QLQ-C30 - European Organisation for Research and Treatment of Cancer Quality of Life Core-30. FACT-G – Functional Assessment of Cancer Therapy – General. MCID – Minimum Clinically Important Difference. RUCC – Rural-Urban Continuum Codes: RUCC 7 Nonmetro county with urban population of 2,500-19,999, not adjacent to a metro area. RUCC 8 Nonmetro county completely rural or less than 2,500 urban population, adjacent. to metro area. RUCC 9 Nonmetro county completely rural or less than 2,500 urban population, not adjacent to metro area. TCI – Threshold for Clinical Importance.

**Table 3e: Rural cancer survivors’ functional health-related quality of life compared with normative and clinically significant values (n=6 studies)**

| **STUDY CHARACTERISTICS** | | | **FUNCTIONAL HEALTH-RELATED QUALITY OF LIFE** | | | | |
| --- | --- | --- | --- | --- | --- | --- | --- |
|  |  |  | **STATISTICAL SIGNIFICANCE** | | **CLINICAL IMPORTANCE** | | |
| **HRQOL INSTRUMENT (SCORING RANGE)**  **(Reference number)** **AUTHOR (YEAR)**  **BY CANCER TYPE(S)** | | **RESIDENTIAL LOCATION** | **STUDY**  **MEAN (95% CI)** | **NORMATIVE**  **MEAN (95% CI)** | **DIFFERENCE (STUDY MEAN - NORMATIVE MEAN)** | **ANCHORS** | **DIRECTION**^§^ |
| **FACT-G (0-28)** | | | **p<0.05** | **p<0.05** |  | **≥2 MCID POINTS**^◊^ |  |
| Breast | (36) DiSipio et al. (2009) | Regional | 21.7 (20.9, 22.5) | 20.2 (19.7, 20.8)* | 1.5 | X | <> |
|  |  | Rural | 21.9 (20.9, 22.9) | 20.2 (19.7, 20.8)* | 1.7 | X | <> |
|  | (47) Cahir et al. (2017) | Rural | 22.0 (18.0, 26.0)^†^ | 19.9 (14.0, 23.3)^†^ | 2.1 | 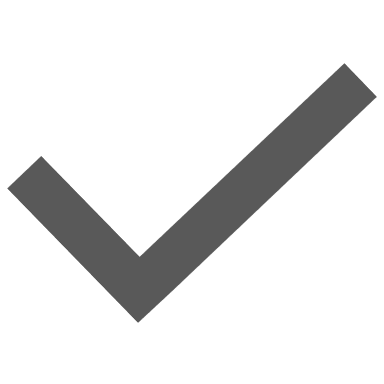 | + |
|  | (44) Santoyo-Olsson et al. (2023) | Rural | 18.2 (17.4, 19.0) | 18.5 (17.9, 19.1) | -0.3 | X | <> |
|  | (48) Vallance et al. (2012) | Rural | 22.6 (22.2, 23.0) | 18.5 (17.9, 19.1)* | 4.1 | 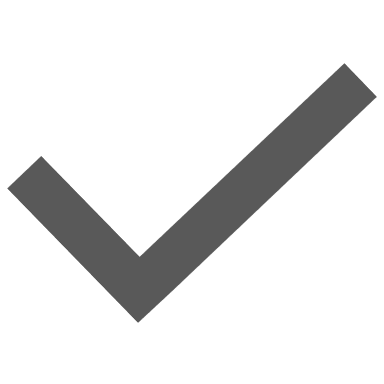 | + |
| Mixed | (43) Strayhorn et al. (2020) | Rural | 19.5 (18.3, 20.7) | 18.5 (17.9, 19.1) | 1.0 | X | <> |
| Head and Neck | (46) Thomas et al. (2014) | Rural | 22.0 (16.0, 26.6) ^Mdn^ | 19.9 (14.0, 23.3) ^Mdn^ | 2.1 | 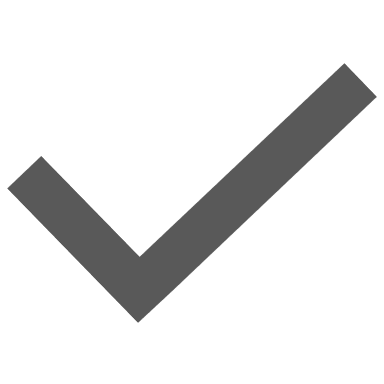 | + |

* Statistically significant difference between study and normative mean; i.e. no cross-over in study and normative 95% confidence intervals.

^◊^ X Not clinically significant; difference between study mean score and normative mean score is <MCID value.
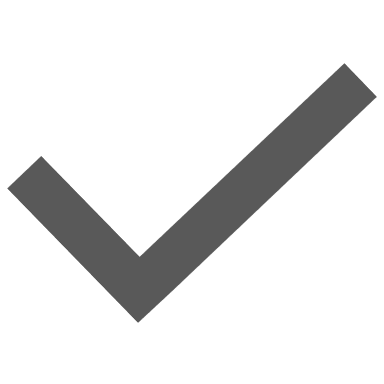
 Clinically significant; difference between study mean score and normative mean score is ≥MCID value.

^§^ <> No clinical difference in HRQOL between study population and normative population (yellow shading). + clinically better HRQOL in the study population than the normative population (green shading).

^Mdn^ Median (Interquartile range) instead of mean (95% CI).

CI – Confidence Interval. FACT-G – Functional Assessment of Cancer Therapy – General. MCID – Minimum Clinically Important Difference.

**Table 3f: Rural cancer survivors’ role and cognitive health-related quality of life compared with normative and clinically significant values (n=1 study)**

| **STUDY CHARACTERISTICS** | | | **STATISTICAL SIGNIFICANCE** | | **CLINICAL IMPORTANCE** | | |
| --- | --- | --- | --- | --- | --- | --- | --- |
| **HRQOL INSTRUMENT**  **(SCORING RANGE)**  **(Reference number)** **AUTHOR (YEAR)**  **BY CANCER TYPE(S)** | | **RESIDENTIAL LOCATION** | **STUDY**  **MEAN (95% CI)** | **NORMATIVE**  **MEAN (95% CI)** | **DIFFERENCE (STUDY MEAN - NORMATIVE MEAN)** | **ANCHORS** | **DIRECTION**^§^ |
| **EORTC QLQ-C30 (0-100)** | | | **ROLE HEALTH-RELATED QUALITY OF LIFE** | | | | |
|  |  |  | **p<0.05** | **p<0.05** |  | **<58 TCI^□^** |  |
| Mixed | (42) Pedro et al. (2014) | RUCC 7 | 83.7 (77.2, 90.2) | 70.5 (70.1, 70.9)* | 13.2 | X | + |
|  |  | RUCC 8 | 85.7 (79.5, 91.9) | 70.5 (70.1, 70.9)* | 15.2 | X | + |
|  |  | RUCC 9 | 85.7 (71.2, 100.2) | 70.5 (70.1, 70.9)* | 15.2 | X | + |
|  |  |  | **COGNITIVE HEALTH-RELATED QUALITY OF LIFE** | | | | |
|  |  |  | **p<0.05** | **p<0.05** |  | **<75 TCI^□^** |  |
|  |  | RUCC 7 | 78.6 (72.0, 85.2) | 82.6 (82.3, 82.9)* | -4.0 | X | + |
|  |  | RUCC 8 | 83.3 (76.4, 90.3) | 82.6 (82.3, 82.9)* | 0.7 | X | + |
|  |  | RUCC 9 | 83.3 (77.4, 89.3) | 82.6 (82.3, 82.9)* | 0.7 | X | + |

* Statistically significant difference between study and normative mean; i.e. no cross-over in study and normative 95% confidence intervals.

**^□^** X Not clinically important; the mean study score is above the TCI value.

^§^ + clinically better HRQOL in the study population than the normative population (green shading).

CI – Confidence Interval. EORTC QLQ-C30 - European Organisation for Research and Treatment of Cancer Quality of Life Core-30. RUCC – Rural-Urban Continuum Codes: RUCC 7 Nonmetro county with urban population of 2,500-19,999, not adjacent to a metro area. RUCC 8 Nonmetro county completely rural or less than 2,500 urban population, adjacent. to metro area. RUCC 9 Nonmetro county completely rural or less than 2,500 urban population, not adjacent to metro area. TCI – Threshold for Clinical Importance.

**Supplementary material 4: Studies of rural cancer survivors’ health-related quality of life without normative and clinically significant values (n= 4 studies)**

| **HRQOL INSTRUMENT**  **(Reference number)** **AUTHOR (YEAR)**  **BY CANCER TYPE(S)** | | **RESIDENTIAL LOCATION** | **MEAN (STANDARD DEVIATION) SCORES** | | | | | |
| --- | --- | --- | --- | --- | --- | --- | --- | --- |
| **QOL-Breast Cancer** § | | | **Physical**  **(0-10)** | **Psychological**  **(0-10)** | **Spiritual**  **(0-10)** |  |  | **Overall**  **(0-10)** |
| Breast | (49) Azam et al 2021 | Rural | 3.08 (0.61) | 3.24 (0.72) | 3.16 (0.71) |  |  | 2.93 (0.92) |
| **QOL-BCS (2005)** † | | |  |  |  |  |  | **Global**  **(0-10)** |
| Breast | (39) Meneses et al 2009 | Rural-Control Arm |  |  |  |  |  | 3.10 (1.14) |
|  |  | Rural-Intervention Arm |  |  |  |  |  | 3.35 (1.63) |
| **QOL-BCS (2008)** † | | |  |  |  |  |  |  |
| Breast | (40) Meneses et al 2020 | Rural-Group A ^¶^ |  |  |  |  |  | 3.16 (1.58) |
|  |  | Rural-Group B ^¶^ |  |  |  |  |  | 3.12 (1.52) |
| **QLAC** ‡ | | | **Appearance related concerns**  **(maximum 20)** | **Benefits of cancer**  **(maximum 20)** | **Family related distress**  **(maximum 15)** | **Recurrence related distress**  **(maximum 20)** | **Financial problems**  **(maximum 20)** |  |
| Mixed | (45) Mandaliya et al 2016 | Rural | 9 (5)  8 (4-12)ˆ | 15 (4)  15 (13-17)ˆ | 9 (4)  9 (7-11)ˆ | 12 (5)  11 (8-17)ˆ | 8 (5)  6 (5-11)ˆ |  |

§ A higher score on the QOL-Breast Cancer represented better HRQOL. † A lower score on the QOL-BCS represented better HRQOL. ‡ A higher score in the QLACS domain ‘benefit’ indicated better QOL but a higher score on the ‘appearance’, ‘family’, ‘recurrence’ and ‘financial’ domains indicated lower QOL. ^¶^ Rural Group A received early education and support. Rural Group B received Support and delayed education.

ˆ Median (Interquartile Range)

QLACS: Quality of Life in Adult Cancer Survivors tool. QOL – Breast Cancer: Quality of Life – Breast Cancer. QOL-BCS: Quality of Life Breast Cancer Survivors.

**Supplementary material 5: Rural-Urban cancer survivors’ health-related quality of life (HRQOL) compared with normative and clinically significant values (n=6 studies)**

| **STUDY CHARACTERISTICS** | | **GLOBAL**  **HEALTH-RELATED QUALITY OF LIFE**  **(Scoring range 0-108)** | | | **PHYSICAL**  **HEALTH-RELATED QUALITY OF LIFE**  **(Scoring range 0-28)** | | | **EMOTIONAL OR/ MENTAL**  **HEALTH-RELATED QUALITY OF LIFE**  **(Scoring range 0-24)** | | | **SOCIAL/FAMILY**  **HEALTH-RELATED QUALITY OF LIFE**  **(Scoring range 0-28)** | | | **FUNCTIONAL**  **HEALTH-RELATED QUALITY OF LIFE**  **(Scoring range 0-28)** | | |
| --- | --- | --- | --- | --- | --- | --- | --- | --- | --- | --- | --- | --- | --- | --- | --- | --- |
| **HRQOL INSTRUMENT,**  **(Reference number) AUTHOR/S (YEAR)** | **RESIDENTIAL LOCATION** | **STUDY MEAN (95% CI)** | **NORMATIVE MEAN (95% CI)** | **DIFFERENCE (STUDY MEAN** - **NORMATIVE MEAN)** | **STUDY MEAN (95% CI)** | **NORMATIVE MEAN (95% CI)** | **DIFFERENCE (STUDY MEAN** - **NORMATIVE MEAN)** | **STUDY MEAN (95% CI)** | **NORMATIVE MEAN (95% CI)** | **DIFFERENCE (STUDY MEAN - NORMATIVE MEAN)** | **STUDY MEAN (95% CI)** | **NORMATIVE MEAN (95% CI)** | **DIFFERENCE (STUDY MEAN** - **NORMATIVE MEAN)** | **STUDY MEAN (95% CI)** | **NORMATIVE MEAN (95% CI)** | **DIFFERENCE (STUDY MEAN** - **NORMATIVE MEAN)** |
| **FACT-G** | |  | | **MCID anchor**  **5 POINTS** |  | | **MCID anchor**  **2 POINTS** |  | | **MCID anchor**  **2 POINTS** |  | | **MCID anchor**  **2 POINTS** |  | | **MCID anchor**  **2 POINTS** |
| (31) DiSipio et al. (2010) | Rural | 86.3 (84.7-88.0) | 85.8 (84.4-87.3) | 0.5 | 22.8 (22.3-23.3) | 25.1 (24.7-25.5) | -2.3 | 19.2 (18.7-19.6) | 20.9 (20.5-21.2) | -1.7 | 22.5 (21.8-23.1) | 19.6 (19.0-20.2) | 2.9 | 21.7 (21.1-22.3) | 20.2 (19.7-20.8) | 1.5 |
|  | Urban | 88.0 (86.3-89.8) | 86.9 (85.8-88.0) | 1.1 | 24.2 (23.8-24.6) | 25.0 (24.8-25.3) | -0.8 | 19.6 (19.1-20.0) | 21.1 (20.8-21.4) | -1.5 | 22.4 (21.6-23.2) | 19.9 (19.5-20.4) | 2.5 | 22.0 (21.3-22.7) | 20.6 (20.2-21.1) | 1.4 |
| (47) Cahir et al. (2017) | Rural | - | - | - | 25.0 (21.0-27.0)^†^ | 24.5 (21.0, 26.8)^†^ | 0.5 | 20.0 (18.0-23.0)^†^ | 21.0 (18.0-24.0)^†^ | -1.0 | 22.0 (17.0-26.0)^†^ | 19.6 (14.0-24.5)^†^ | 2.4 | 22.0 (18.0-26.0)^†^ | 19.9 (14.0-23.3)^†^ | 2.1 |
|  | Urban | - | - | - | 24.0 (20.0-27.0) ^†^ | 24.5 (21.0, 26.8)^†^ | -0.5 | 20.0 (17.0-22.0)^†^ | 21.0 (18.0-24.0)^†^ | -1.0 | 22.0 (17.0-26.0) ^†^ | 19.6 (14.0-24.5)^†^ | 2.4 | 21.0 (17.0-25.0)^†^ | 19.9 (14.0-23.3)^†^ | 1.1 |
| (44) Santoyo-Olsson et al. (2023) | Rural | 74.0 (71.5, 76.5) | 80.1 (78.6, 81.6) | -6.1 | 20.4 (19.4-21.4) | 22.7 (22.4, 23.0) | -2.3 | 17.3 (16.6-18.0) | 19.9 (19.6-20.2) | -2.6 | 18.2 (17.4-19.0) | 19.1 (18.7-19.5) | -0.9 | 18.2 (17.4-19.0) | 18.5 (17.9-19.1) | -0.3 |
|  | Urban | 66.1 (63.5-68.7) | 80.1 (78.6-81.6) | -14.0 | 18.6 (17.6-19.6) | 22.7 (22.4-23.0) | -4.1 | 14.9 (14.0-15.8) | 19.9 (19.6-20.2) | -5.0 | 17.3 (16.4-18.2) | 19.1 (18.7-19.5) | -1.8 | 15.2 (14.3-16.1) | 18.5 (17.9-19.1) | -3.3 |
| (46) Thomas et al. (2014) | Rural | - | - | - | 25.0 (21.0-28.0)^†^ | 24.5 (21.0, 26.8)^†^ | 0.5 | 21.0 (19.0-23.0)^†^ | 21.0 (18.0-24.0)^†^ | 0 | 22.2 (18.0-25.7)^†^ | 19.6 (14.0-24.5)^†^ | 2.6 | 22.0 (16.0-26.6)^†^ | 19.9 (14.0-23.3)^†^ | 2.1 |
|  | Urban | - | - | - | 25.0 (19.0-28.0) ^†^ | 24.5 (21.0, 26.8)^†^ | 0.5 | 20.0 (17.0-23.0) | 21.0 (18.0-24.0)^†^ | -1.0 | 21.0 (16.3-25.7) ^†^ | 19.6 (14.0-24.5)^†^ | 1.4 | 20.0 (15.0-25.0)^†^ | 19.9 (14.0-23.3)^†^ | 0.1 |
|  | |  | | | **PHYSICAL**  **HEALTH-RELATED QUALITY OF LIFE**  **(Scoring range 0-100)** | | | **MENTAL**  **HEALTH-RELATED QUALITY OF LIFE**  **(Scoring range 0-100)** | | |  | | |  | | |
| **SF-36** | |  | | |  | | **MCID anchor**  **5 POINTS** |  | | **MCID anchor**  **5 POINTS** |  | | |  | | |
| (38) Gray et al. (2019) | Rural | -- | -- | -- | 43.7 (42.4, 45.0) | 50.0 (49.8, 50.2) | -6.3 | 56.7 (55.6, 57.8) | 50.0 (49.8, 50.2) | 6.7 | -- | -- | -- | -- | -- | -- |
|  | Urban | -- | -- | -- | 44.7 (43.6-45.8) | 50.0 (49.8, 50.2) | -5.3 | 56.5 (55.8-57.2) | 50.0 (49.8, 50.2) | 6.5 | -- | -- | -- | -- | -- | -- |
| (50) Socha et al. (2021) | Rural | -- | -- | -- | 51.7 (44.2, 59.2) | 50.0 (49.8, 50.2) | 1.7 | 53.9 (44.2, 63.6) | 50.0 (49.8, 50.2) | 3.9 | -- | -- | -- | -- | -- | -- |
|  | Urban | -- | -- | -- | 58.7 (56.3-61.1) | 50.0 (49.8, 50.2) | 8.7 | 63.8 (61.2-66.4) | 50.0 (49.8, 50.2) | 13.8 | -- | -- | -- | -- | -- | -- |

^†^ Median (Interquartile range)

-Not reported by study authors

--Not collected by SF-36

CI - Confidence Interval. FACT-G - Functional Assessment of Cancer Therapy-General (FACT-G). MCID - Minimum Clinically Important Difference. SF-36 - Medical Outcomes Study Short-Form 12 or 36. Ref # - Reference number.

**Supplementary material 6: Studies of rural and urban cancer survivors’ health-related quality of life (HRQOL) without normative and clinically significant values (n=4 studies)**

| **HRQOL INSTRUMENT**§  **(Reference number)** **AUTHOR (YEAR)**  **BY CANCER TYPE(S)** | | **RESIDENTIAL LOCATION** | **HRQOL SCORES** | | | | |
| --- | --- | --- | --- | --- | --- | --- | --- |
| **QOL-Breast Cancer** | | | **Physical**  **(O-10)** | **Psychological**  **(0-10)** | **Spiritual**  **(0-10)** |  | **Overall**  **(0-10)** |
| Breast | (49) Azam et al 2021 | Rural | 3.08 (0.61) | 3.24 (0.72) | 3.16 (0.71) |  | 2.93 (0.92)¥ |
|  |  | Urban | 2.79 (0.62) | 3.00 (0.79) | 3.59 (0.64) |  | 3.56 (0.84)¥ |
| **FACT-B+4** | | |  |  |  |  | **Overall**  **(0-160)** |
| Breast | (31) DiSipio et al 2010 | Rural |  |  |  |  | 122.6 (120.3, 125.0)‡ |
|  |  | Urban |  |  |  |  | 130.2 (127,7, 132.7)‡ |
| **FACT-ES** | | |  |  |  |  | **Overall**  **(0-184)** |
| Breast | (47) Cahir et al 2017 | Rural |  |  |  |  | 123 (104-138)† |
|  |  | Urban |  |  |  |  | 126 (107-143)† |
| **FACT-HN** | | |  |  |  | **Head & Neck Cancer Subscale**  **(0-40)** |  |
| Head & Neck | (46) Thomas et al 2014 | Rural |  |  |  | 31.0 (23.0-35.6)† |  |
|  |  | Urban |  |  |  | 29.0 (22.0-35.0)† |  |

§ A higher score on the QOL-Breast Cancer, FACT-B+4, FACT-ES and FACT-HN represented better HRQOL. ¥ Mean (Standard Deviation) ‡ Mean (95% Confidence Interval). † Median (Interquartile Range). FACT-B+4: Functional Assessment of Cancer Therapy – Lymphedema. FACT-ES: Functional Assessment of Cancer Therapy – Endocrine Symptoms. FACT-HN: Functional Assessment of Cancer Therapy – Head and Neck. QOL – Breast Cancer: Quality of Life – Breast Cancer
